# Magnetic Microrobots for Drug Delivery: A Review of Fabrication Materials, Structure Designs and Drug Delivery Strategies

**DOI:** 10.3390/molecules31010086

**Published:** 2025-12-25

**Authors:** Jin Shi, Yanfang Li, Dingran Dong, Junyang Li, Tao Wen, Yue Tang, Qi Zhang, Fei Pan, Liqi Yan, Duanpo Wu, Shaowei Jiang

**Affiliations:** 1 School of Automation, Hangzhou Dianzi University, Qiantang District, Hangzhou 310020, China; 242060290@hdu.edu.cn; 2 School of Communication Engineering, Hangzhou Dianzi University, Qiantang District, Hangzhou 310020, China; 3 School of Computer Science, Hangzhou Dianzi University, Qiantang District, Hangzhou 310020, China; 4 Department of Electronic Engineering, Ocean University of China, Qingdao 266100, China; 5 Shandong Key Laboratory of Intelligent Sensing Chip and System, Qingdao 266100, China; 6 North Automatic Control Technology Institute, Taiyuan 030006, China; 7 School of Integrated Circuits and Electronics, Beijing Institute of Technology, Beijing 100081, China; 8 Chongqing Institute of Microelectronics and Microsystems, Beijing Institute of Technology, Chongqing 400030, China; 9 Department of Biomedical Engineering, City University of Hong Kong, Hong Kong SAR 999077, China; qi.zhang@my.cityu.edu.hk; 10 Hong Kong Center for Cerebro-Cardiovascular Health Engineering (COCHE), Hong Kong SAR 999077, China; 11 School of Interdisciplinary Studies, Lingnan University, Tuen Mun, New Territories, Hong Kong SAR 999077, China

**Keywords:** drug delivery, magnetic microrobots, drug loading, drug release

## Abstract

Magnetic microrobots have emerged as a promising platform for drug delivery in recent years. By enabling remotely controlled motion and precise navigation under external magnetic fields, these systems offer new solutions to overcome the limitations of traditional drug delivery nanocarriers, such as inadequate tissue penetration and heterogeneous biodistribution. Over the past few years, significant advancements have been made in the structural design of magnetic microrobots, as well as in drug loading techniques and stimuli-responsive drug release mechanisms, thereby demonstrating distinct advantages in enhancing therapeutic efficacy and targeting precision. This review provides a comprehensive overview of magnetic drug delivery microrobots, which are categorised into biomimetic structural, bio-templated and advanced material-based types, and introduces their differences in propulsion efficiency and biocompatibility. Additionally, drug loading and release strategies are summarised, including physical adsorption, covalent coupling, encapsulation, and multistimuli-responsive mechanisms such as pH, enzyme activity and thermal triggers. Overall, these advancements highlight the significant potential of magnetic microrobots in targeted drug delivery and emphasise the key challenges in their clinical translation, such as biological safety, large-scale production and precise targeted navigation within complex biological environments.

## 1. Introduction

The growing demand for precision medicine and personalised treatments in modern healthcare has made efficient, controllable and low-toxicity drug delivery a critical challenge in biomedical research [1,2,3]. Traditional drug administration methods, such as oral [4], intravenous [5] or transdermal delivery [6], remain the most commonly used in clinical practice because of their simplicity and ease of application. However, these approaches suffer from several critical limitations, including poor targeting ability, fluctuating drug concentrations and systemic side effects. To overcome these issues, researchers have developed a variety of nanocarrier-based drug delivery systems, including liposomes [7], polymeric nanoparticles [8], dendrimers [9], inorganic nanoparticles [10], pollen-based microparticles [11], and microalgae [12]. These systems have shown promise in improving pharmacokinetic profiles, enhancing tissue accumulation and enabling controlled drug release. However, a fundamental limitation of these nanocarriers is their reliance on passive diffusion and systemic circulation, which often leads to limited tissue penetration, uneven biodistribution, and premature drug leakage [13,14]. In order to overcome the limitations of passive transportation, microrobots were born as a promising strategy that can actively propel and accurately navigate [15,16,17]. Unlike passive carriers, microrobots can convert various energy sources into kinetic energy to overcome hemodynamic resistance and penetrate complex biological barriers. At present, active promotion is mainly achieved through a variety of driving strategies, including chemical [18], bio-hybrid [19], and physical field actuation [20,21]. Although significant progress has been made in these areas, their clinical translation still faces substantial bottlenecks. For instance, chemical propulsion often relies on toxic fuels, resulting in poor biocompatibility. Similarly, optical and electrical actuation methods suffer from extremely limited penetration depths within biological tissues. While acoustic actuation offers deep tissue penetration, achieving precise control in complex environments remains a challenge. In contrast, magnetic microrobots represent a superior alternative for deep-tissue applications due to their unique actuation mechanism and safety profile. Magnetic microrobots typically comprise a magnetic substance (e.g., iron oxide or nickel) embedded within the structures, which can respond to external magnetic fields generated by electromagnetic coils or permanent magnets. By modulating the gradient and direction of the external field, magnetic torques and pulling forces are exerted on the microrobot, converting magnetic energy into mechanical locomotion. This enables microrobots to achieve a variety of movement modes, such as rolling, tumbling or spiral swimming, so that they can accurately navigate in the sticky fluid and blood vessel network without carrying fuel. In addition, the magnetic field can penetrate human tissue with extremely small attenuation and is non-toxic, and the technology itself is compatible with medical imaging methods such as magnetic resonance imaging [22,23]. Consequently, magnetic microrobots have attracted significant research interest in the field of targeted drug delivery.

Over the last few years, research on magnetic microrobots for drug delivery has gradually transitioned from primarily enhancing movement capabilities to a combined approach that integrates structural design with drug delivery features. Early studies focused on the development of bioinspired structures, including helical [24], rod-like [25], conical [26] and shell-shaped [27] geometries. These designs, which mimic the propulsion mechanisms of microorganisms, demonstrate excellent propulsion efficiency and controllability in low-Reynolds-number environments, laying a solid foundation for microrobot navigation in complex biological fluids [24]. However, their limited surface area constrains payload capacity, limiting therapeutic applicability. To overcome this restriction, researchers have started exploring bio-templated microrobot systems based on organisms such as bacteria [28], macrophages [29], *Spirulina* [30] and *Chlorella* [31]. These natural carriers offer large surface areas, enhanced biocompatibility, and specific chemotactic abilities [32]. Drugs can be loaded through physical encapsulation or covalent modification and then released in response to pH or enzymatic conditions at diseased sites [33]. This approach greatly enhances in vivo stability and therapeutic efficacy. However, challenges such as large-scale fabrication and immune evasion still limit their translational potential. At the same time, the introduction of stimulus-responsive materials has opened up a new way to expand the functions of microrobots. For example, pH-responsive polymers (such as chitosan) have been used to trigger drug release in the acidic tumour microenvironment [34]. Heat-responsive hydrogels (such as poly(N-isopropyl acrylamide) (PNIPAM)) use body temperature or heat therapy to control the payload capacity [35]. And light-responsive liquid crystal elastomers (LCEs) can provide deformation for active cargo transport and manipulation [36]. In recent years, hydrogels [37] and metal–organic frameworks (MOFs) [38] have been extensively utilised in the fabrication of microrobots. Hydrogels have been extensively used in the design of magnetic microrobots due to their excellent biocompatibility and degradability. These microrobots can achieve high encapsulation rates through encapsulation, enabling selective release in response to a change in pH or temperature. Meanwhile, embedded magnetic nanoparticles enable remote control, allowing the synchronised management of movement and release profiles [39,40]. Compared with bioinspired or bio-templated systems, hydrogel-based magnetic microrobots exhibit advantages in cargo carrying performance and release accuracy.

This review systematically summarises recent advances in magnetic microrobots used for drug delivery, highlighting the unique advantages of magnetic actuation in this field. Firstly, we classify and analyse three types of magnetic microrobots based on their structural designs: bioinspired, bio-templated and advanced material-based systems, and compare various drug loading strategies, such as physical adsorption, covalent conjugation and encapsulation. Secondly, we summarise various release mechanisms, including responses to pH, enzymes and temperature, as well as external stimuli such as magnetic fields, light, ultrasound and multistimuli-responsive systems. Their respective advantages and limitations are discussed. Finally, we outline the significant challenges facing magnetic microrobots in targeted drug delivery and offer perspectives on future research directions.

## 2. Classification of Magnetic Microrobots

Magnetic microrobots exhibit considerable diversity, demonstrating notable differences in their structural characteristics, material composition and functional capabilities. Different types of microrobots not only have distinct characteristics in motion performance and biocompatibility, but their structural design also directly influences the drug loading capacity and subsequent release mechanisms.

### 2.1. Magnetic Microrobots Based on Biological Templates

Magnetic microrobots based on biological templates are defined as biohybrid systems that utilise naturally occurring biological entities, such as cells, bacteria, or microalgae, as the primary structural scaffold [41]. By integrating magnetic particles with these natural structures, this approach synergizes innate biological functions with external magnetic control capabilities, thereby offering exceptional biocompatibility and efficient self-propulsion within complex physiological environments [42].

#### 2.1.1. Cell-Based Magnetic Microrobots

Cell-based magnetic microrobots utilise natural cells as structural templates, integrating innate biological functions with externally added magnetic control systems [43]. These systems exhibit high biocompatibility, active targeting capability and adaptability to complex biological environments [29,44]. Common cell templates include stem cells, immune cells, bacteria, and erythrocytes [45,46,47]. Cells can be magnetised by internalising or attaching magnetic nanoparticles to their surfaces, enabling them to move under magnetic fields [48]. Their native membrane and cytoplasmic structures provide natural cargo capacity, effectively protecting therapeutic payloads from degradation and immune clearance while preserving drug activity [49,50].

**Figure 1:**
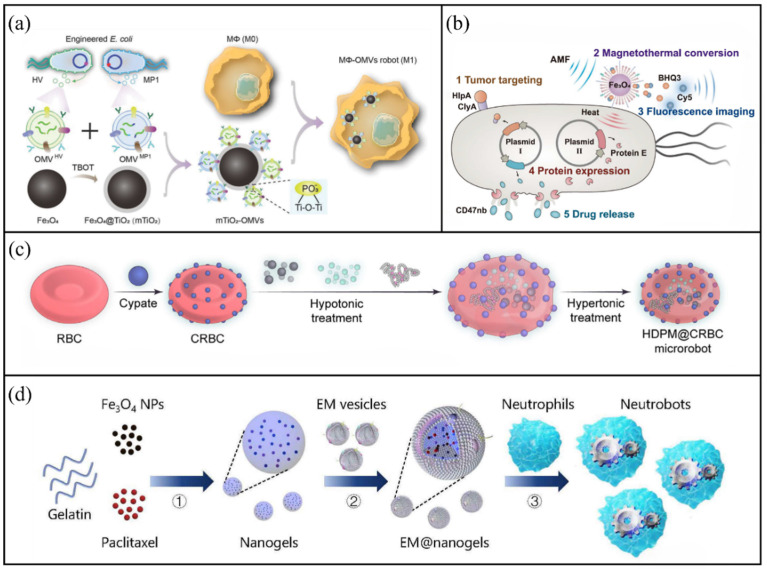
Cell-based microrobots. (**a**) Schematic of MΦ-OMV microrobot preparation. Reproduced with permission from Ref. [51]. Copyright © 2023, IEEE. (**b**) Composition and design of the AMF-Bac system. Reproduced with permission from Ref. [53]. Copyright © 2023, Open access. (**c**) Schematic diagram of MCR preparation. Reproduced with permission from Ref. [46]. Copyright 2023, Open access. (**d**) Schematic illustration of the fabrication process of a neutrophil-based microrobot. Reproduced with permission from Ref. [62]. Copyright © 2021, The American Association for the Advancement of Science.

Macrophages are frequently used as carriers for cell-based microrobots because of their innate phagocytic activity and tumour-targeting ability. In addition, their excellent biocompatibility enables them to efficiently internalise magnetic nanoparticles, allowing directional movement and targeted delivery under the control of an external magnetic field. Sun et al. developed a magnetically controlled cell robot (MCRs) based on macrophages [51]. This MCRs was loaded with multiple cytokines (IL-12, CCL-5 and CXCL-10). After loading, the macrophages presented a distinct M1 polarisation phenotype, thereby possessing the inherent potential to inhibit tumour growth and regulate the tumour microenvironment. By optimising the concentration of PLL@FeNPs (ideally 40 μg/mL), the study ensured that MCRs had good biocompatibility and low toxicity while maintaining high cell viability. Based on this, Li et al. further innovated and proposed a multimodal cancer therapy microrobot system based on macrophages [52]. As shown in Figure 1a, genetically engineered bacterial outer membrane vesicles (OMVs) were immobilised on the surface of magnetic nanoparticles and internalised by macrophages to construct the MΦ-OMV microrobot. This layered assembly design not only endows the system with magnetic controllability, but more importantly, it reprograms macrophages into the M1 state with an anti-tumour phenotype by means of the loaded active components, thereby simultaneously achieving the dual effects of magnetic targeted accumulation and immune regulation. This system effectively activates the anti-tumour immune response by integrating the tumour suppressive function of macrophages, the immune stimulating effect of OMVs, and the synergistic effect of anti-tumour peptides, demonstrating the potential of multimodal synergistic treatment. The main advantages of engineered bacterial microrobots lie in their natural tumour homing ability and the precise controllability brought by modular design. Ma et al. proposed a system named alternating magnetic field-manipulated tumour-homing bacteria (AMF-Bac) nanocomposites [53]. As shown in Figure 1b, anti-cd47 nanobodies (CD47nb) are pre-expressed and stored within bacteria. Fe_3_O_4_ nanoparticles act as “signal decoding” modules, converting alternating magnetic field (AMF) signals into thermal signals, thereby initiating the expression of bacterial lysis proteins controlled by thermosensitive starters, ultimately leading to bacterial lysis and the release of CD47nb. The main difference between this drug release mechanism and the immune cell platform lies in the tumour-targeted delivery of CD47nb, which avoids the haematological toxicity brought by traditional antibodies, thereby enhancing therapeutic efficacy and biological safety.

Although engineered bacteria can provide precise control, their clinical translation still faces safety challenges. For applications that require excellent biocompatibility and prolonged systemic circulation, red blood cells (RBCS) are an ideal carrier due to their relatively simple structure, large inner cavity, and long circulation time in the body [54,55,56]. Zhu et al. proposed a red blood cell-derived microrobot for precise, controllable and hierarchical drug delivery [46]. As shown in Figure 1c, near-infrared (NIR)-responsive cypate dye was covalently attached to the red blood cell membrane, while magnetic nanoparticles, an albumin–doxorubicin complex (DOX@HSA) and pirfenidone (PFD) were co-loaded on the microrobot surface to fabricate the HDPM@CRBC system. This study proposes that the loaded PFD can inhibit the formation of extracellular matrix (ECM), thereby enhancing the deep penetration efficiency of DOX@HSA in solid tumours and ultimately effectively suppressing both primary and metastatic tumours. During inflammation, neutrophils (NEs) migrate along chemokine gradients across the BBB or blood-brain tumour barrier [57,58]. Using this property, NEs have been engineered to serve as drug carriers for targeting inflammatory tumours [59,60,61]. Zhang et al. developed an NE-based microrobot capable of actively delivering drugs to malignant glioma sites in vivo [62]. As shown in Figure 1d, these microrobots were fabricated through the process of natural NEs phagocytosing drug-encapsulated magnetic nanogels coated with *E. coli* membranes.

In short, cell-based magnetic microrobots take advantage of the natural advantages of cells (such as macrophages, bacteria, red blood cells and neutrophils), especially their biocompatibility and drug-carrying capacity. This biological mixing method combines external magnetic control and biological tendency, so that drugs can be effectively delivered outside complex biological barriers.

#### 2.1.2. Magnetic Microrobots Based on Microalgae

Magnetic microalgae-based microrobots utilise the natural structures and biological functions of unicellular algae, such as diatoms and green algae, to combine photosynthesis, unique cell wall shapes and excellent biocompatibility with an external magnetic control system [12,63,64]. This combination enables the creation of small systems capable of active propulsion and multifunctional therapy. The cell walls of many microalgae species contain porous silica-based or polysaccharide-based structures that permit the loading of various therapeutic agents and enable photo-triggered activation [65,66].

**Figure 2:**
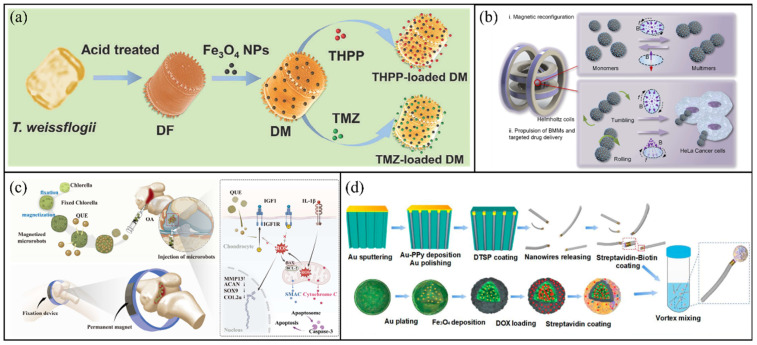
Microalgae-based microrobots. (**a**) Schematic illustration of the fabrication process of a microalgal microshell-based microrobot. Reproduced with permission from Ref. [68]. Copyright © 2024, Wiley-VCH GmbH. (**b**) Magnetic reconfiguration and propulsion of the microrobot. Reproduced with permission from Ref. [31]. Copyright © 2022, American Chemical Society. (**c**) Therapeutic mechanism of M-CH@QUE for osteoarthritis treatment. Reproduced with permission from Ref. [69]. Copyright © 2025, Wiley-VCH GmbH. (**d**) Preparation of drug-loaded BFSMs. Reproduced with permission from Ref. [71]. Copyright © 2024, American Chemical Society.

Diatoms and microalgae have recently garnered increased attention as natural bio-templates for constructing magnetic microrobots, because of their inherent biocompatibility, large surface-to-volume ratio and structural versatility [67]. Li et al. reported a diatom-based magnetic microrobot platform for treating deep-seated glioblastoma (GBM) [68]. As shown in Figure 2a, natural porous silica frustules from diatoms were employed to adsorb chemotherapeutic drugs and photosensitisers physically. This platform has achieved a synergistic treatment with dual drugs and dual release mechanisms. The chemotherapy drug temozolomide (TMZ) is ph-sensitive and has the characteristics of long-term slow release to enable continuous chemotherapy. The photosensitizer 5,10,15,20-Tetrakis(4-hydroxyphenyl)porphyrin(THPP) is loaded in the cavity and triggered by ultrasonic vibration to break the diatom structure, achieving explosive release. *Chlorella* has also been extensively used as a bio-template because of its large cell surface and strong biocompatibility. Gong et al. developed biohybrid microrobot multimers (BMMs) based on magnetised *Chlorella* cells [31]. As illustrated in Figure 2b, these microrobots exhibit dynamic structural changes and multimodal movement, including rolling and tumbling, driven by external magnetic fields. Notably, BMMs show outstanding propulsion and control in low Reynolds number conditions, with rolling dimers attaining speeds of up to 107.6 μm/s in a 70 Gs processing magnetic field. In addition to propulsion efficiency, the *Chlorella* platform is also used to address delivery challenges in the treatment of specific diseases, focusing on the accumulation and long-lasting release of drugs. Chen et al. introduced M-CH@QUE microrobots, where magnetised *Chlorella* cells were loaded with quercetin for treating anteromedial osteoarthritis (AMOA) [69]. As shown in Figure 2c, this microrobot can be guided to focus on the lesion area of anterior medial osteoarthritis (AMOA) through an external magnetic field, thereby significantly reducing the frequency and concentration of intra-articular injection, overcoming the limitations of traditional intra-articular injection methods, and providing a new treatment approach for early osteoarthritis.

Additionally, researchers are working to enhance the structural and functional diversity of microrobots derived from *Chlorella* [32,70]. Celi et al. developed biohybrid flexible sperm-like microrobots (BFSMs) by combining *Chlorella* heads with artificial flagella made of polypyrrole nanowires [71]. As shown in Figure 2d, the head of the drug-loaded magnetic *Chlorella* was connected to the tail of the artificially synthesised flexible pyrrole nanowires (PPy nanowires) through biotin-streptavidin bonding, and the feasibility of using flexible sperm-like microrobots for targeted drug delivery was verified for the first time. Building on this, Celi et al. also described a scalable method for creating AlgaeSperm microrobots, utilising magnetic-assisted in situ polymerisation to attach flexible magnetic tails onto magnetised *Chlorella* heads [72]. These microrobots exhibit strong propulsion, reaching speeds of up to 2.3 body lengths per second, making them the fastest known sperm-like magnetic microrobots to date.

In summary, magnetic microrobots based on microalgae leverage the unique morphological features and biological functions of natural algae, such as the porous silica frustules of diatoms and the robust cellular structure of *Chlorella*. By integrating these bio-templates with magnetic actuation, these systems achieve versatile locomotion modes, ranging from collective swarm behaviours to sperm-like swimming, while offering high drug-loading capacities and intrinsic photothermal properties.

### 2.2. Magnetic Microrobots Based on Advanced Material

Although biological template microrobots exhibit excellent biocompatibility, challenges persist regarding their large-scale manufacturing and reproducibility. To address these limitations, magnetic microrobots based on advanced materials have emerged as a robust alternative. These systems are defined by their reliance on intrinsic physicochemical properties, such as the tuneable porosity of metal–organic frameworks (MOFs) or the stimuli-responsive swelling of hydrogels, as the primary mechanism for drug loading and release.

#### 2.2.1. Magnetic Hydrogel-Based Microrobots

Hydrogels, comprising 3D networks of hydrophilic polymer chains, have become exemplary materials for the development of intelligent magnetic microrobots because of their superior biocompatibility, high water content, adjustable mechanical properties and biodegradability [73,74]. The incorporation of magnetic nanoparticles into hydrogel matrices facilitates remote magnetic actuation and precise navigation [75,76]. Furthermore, hydrogels can be chemically modified or physically loaded with therapeutic agents to confer multiple stimulus-responsive functionalities, thereby enabling controlled drug release at target sites [77,78].

**Figure 3:**
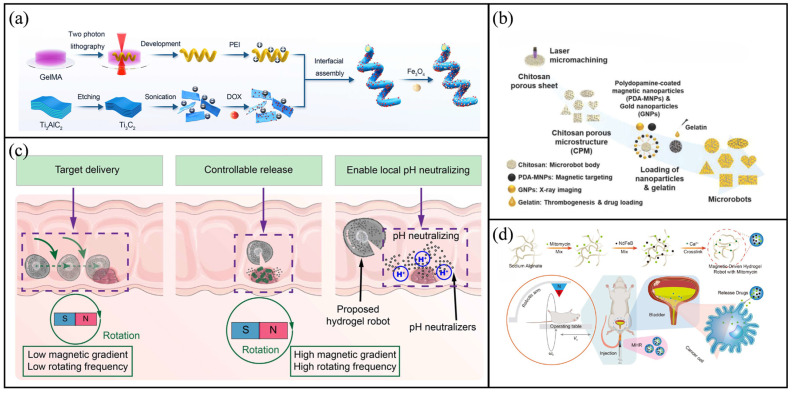
Hydrogel-based microrobots. (**a**) Schematic of the fabrication process of MXBOTs@DOX. Reproduced with permission from Ref. [82]. Copyright © 2024, American Chemical Society. (**b**) Preparation procedure of shape-specific microrobots via laser micromachining of porous chitosan sheets, nanoparticle attachment and gelatine loading. Reproduced with permission from Ref. [85]. Copyright © 2023, Wiley-VCH GmbH. (**c**) Schematic diagram of oral multilayer hydrogel microrobot drug release. Reproduced with permission from Ref. [88]. Copyright © 2023, Open access. (**d**) Fabrication process of the MMHR. Reproduced with permission from Ref. [89]. Copyright © 2025, American Chemical Society.

Advanced fabrication techniques have significantly broadened the structural and functional diversity of hydrogel-based microrobots. For instance, two-photon polymerization (TPP) has been employed to produce 3D microhelical structures with high precision [79,80,81]. Utilising this methodology, Yang et al. developed multifunctional MXene-based magnetically actuated microrobots (MXBOTs) through the coating of gelatine methacryloyl (GelMA) hydrogel helices with Fe_3_O_4_ nanoparticles and Ti_3_C_2_ MXene loaded with doxorubicin (DOX) nanosheets [82]. As shown in Figure 3a, the MXene coating endows the microrobot with photothermal properties and photoacoustic imaging functionalities, while the hydrogel scaffold provides biodegradability and biocompatibility. These MXBOTs demonstrate drug release under acidic pH and thermal stimuli, achieving synergistic chemo–photothermal therapy, and confirm the feasibility of 2D MXene modification for multifunctional biomedical microrobots.

Microfluidic droplet technology has demonstrated significant benefits for applications that require large quantities of consistently spherical microrobots [83]. Tao et al. documented the development of a magnetically actuated hydrogel microrobot, fabricated through droplet microfluidics, which simultaneously encapsulated DOX and the CDK1 inhibitor Ro-3306 within the hydrogel matrix [84]. This dual-drug delivery system effectively eliminates osteosarcoma cells and significantly enhances the chemosensitivity of MYC-dependent tumours, offering a new approach for targeted combination therapies.

Bio-templating approaches have also been applied to incorporate hydrogel functionalities with natural microstructures. Go et al. developed a biodegradable chitosan-based microrobot for targeted vessel chemoembolisation [85]. As shown in Figure 3b, this microrobot was composed of chitosan with a porous structure, gelatine for encapsulating drugs, magnetic nanoparticles coated with polydopamine (PDA) for achieving magnetic targeting and gold nanoparticles for real-time X-ray visualisation. Notably, the gold nanoparticles provided superior imaging contrast compared to traditional iodine-based agents, thereby facilitating precise image-guided therapy [86]. Ionic crosslinking presents an additional versatile approach for fabricating hydrogel microrobots [87]. Xu et al. developed an oral multilayer hydrogel microrobot intended for gastrointestinal therapy [88]. As shown in Figure 3c, by sequentially encapsulating a pH neutraliser in the outer layer and therapeutic agents in the inner layer, the microrobot achieved staged release under programmed magnetic fields. This multilayer design provided more efficient and precise release compared with conventional oral capsules.

Hydrogel-based microrobots have also demonstrated clinical relevance in bladder cancer therapy. Jia et al. developed mitomycin-loaded magnetic hydrogel microrobots (MHRMs) by combining sodium alginate, NdFeB nanoparticles and mitomycin, followed by Ca^2+^-mediated ionic cross-linking [89]. As shown in Figure 3d, when injected into the bladder through the urethra, the MHRMs were activated magnetically to enable spiral movement along the mucosal wall, ensuring even drug distribution. Compared with traditional intravesical perfusion, this method significantly enhances drug penetration and targeted accuracy, providing a promising alternative for clinical treatment.

In summary, magnetic hydrogel-based microrobots combine the biocompatibility, flexibility, and drug-loading capacity of hydrogels with the precise controllability of magnetic actuation. Through advanced fabrication techniques such as two-photon polymerization, microfluidic droplet synthesis, and bio-templating, these systems achieve programmable architectures and multifunctional therapeutic capabilities. 

#### 2.2.2. Magnetic MOF-Based Microrobots

MOFs, a class of crystalline porous materials composed of metal ions or clusters and organic linkers, have demonstrated significant potential in drug delivery applications due to their exceptionally high surface area, adjustable pore size and facile functionalisation [90]. In the context of magnetic microrobots, MOFs may function not only as high-capacity drug carriers but also as integrated components with magnetic elements to facilitate spatially targeted and regulated drug release [91,92]. Compared with traditional porous materials, MOFs are capable of undergoing stimulus-responsive structural modifications, such as ligand dissociation, framework disintegration or pore opening, in response to external stimuli including acidic microenvironments, specific wavelengths of light or temperature fluctuations [93]. These modifications substantially enhance the selectivity and efficacy of drug delivery processes.

**Figure 4:**
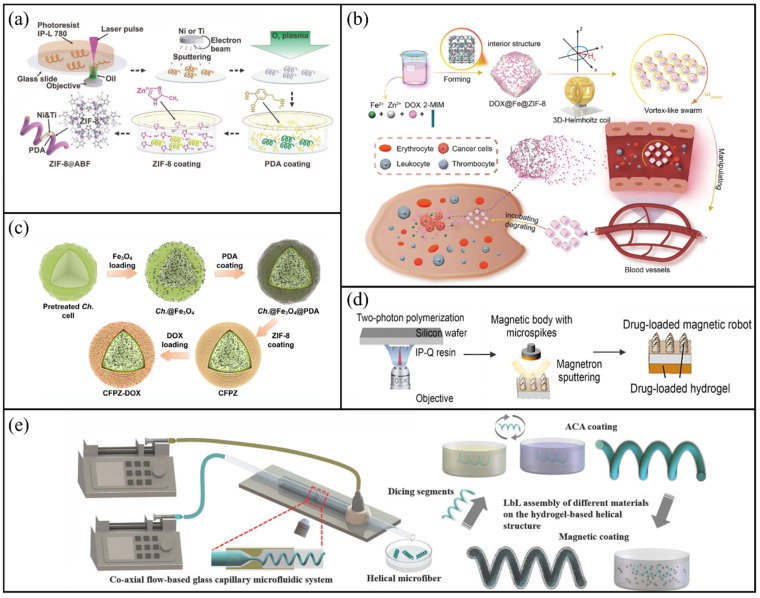
Hydrogel-based and bioinspired microrobots. (**a**) Schematic of the fabrication process for the ZIF-8@ABF microrobot. Reproduced with permission from Ref. [94]. Copyright © 2019, WILEY-VCH Verlag GmbH & Co. KGaA, Weinheim. (**b**) Illustration of the formation and magnetic manipulation of MMRSs under a customised magnetic field, along with pH-responsive drug release. Reproduced with permission from Ref. [95]. Copyright © 2024, Science China Press. (**c**) Preparation scheme of the CFPZ-DOX microrobot. Reproduced with permission from Ref. [96]. Copyright © 2025, American Chemical Society. (**d**) Manufacturing process of the magnetic microspike robot. Reproduced with permission from Ref. [103]. Copyright © 2025, Zhejiang University Press. (**e**) Fabrication and functionalisation of the magnetic helical microrobot. Reproduced with permission from Ref. [105]. Copyright © 2021, Royal Society of Chemistry.

Zeolite imidazolate framework-8 (ZIF-8), a representative example of MOFs, demonstrates remarkable drug-loading capacity and pH-responsive degradation characteristics. Wang et al. developed a biocompatible and pH-responsive magnetic helical microrobot [94]. As shown in Figure 4a, the microhelix was constructed through TPP, functionalised with PDA and finally coated with ZIF-8 crystals grown in situ. This microrobot can be propelled along predefined trajectories under a rotating magnetic field and execute drug delivery tasks inside microfluidic channels. This achievement marks the first demonstration of the potential of MOF-based microrobots for targeted therapeutic applications.

Compared with individual microrobots, magnetic microrobot swarms (MMRs) provide increased cargo capacity, sophisticated navigation capabilities and remarkable environmental adaptability. Cao et al. developed an MOF-based magnetic microrobot swarm for collective drug delivery [95]. As shown in Figure 4b, DOX-loaded MMRs were synthesised via one-pot mineralisation. Under programmable magnetic fields, the swarm can reconfigure into different shapes to adapt to complex environments and be precisely navigated to target sites. The pH-responsive degradation of ZIF-8 within acidic tumour microenvironments (pH = 5.5–6.5) facilitate the release of DOX, resulting in the significant inhibition of T24 tumour cells. In addition, Gu et al. successfully integrated MOF technology with bio-templated magnetic microrobots for the first time, developing a novel *Chlorella*-based system with validated capabilities in magnetic actuation, photothermal conversion, drug delivery, biocompatibility and synergistic therapy in vitro [96]. As shown in Figure 4c, a ZIF-8 layer was synthesised in situ on the surface of the microalgae to establish a porous drug reservoir, with magnetic nanoparticles integrated for accurate navigation. DOX was loaded into the MOF pores and released under acidic conditions or photothermal stimulation. Together with the photothermal effect of the MOF, this system markedly improved the efficacy of chemo-photothermal therapy, providing a novel platform for targeted combination treatment.

In summary, magnetic MOF-based microrobots exploit the high surface area and tuneable porosity of metal–organic frameworks to maximise drug-loading efficiency. Their ability to undergo structural modifications in response to specific biological cues (e.g., acidic tumour microenvironments) or external stimuli (e.g., light and heat) enables precise, on-demand drug release at target sites.

### 2.3. Magnetic Bionic-Structured Microrobots

Although magnetic microrobots made of advanced materials offer advantages such as facile mass production and multifunctional capabilities, their locomotion is often constrained by the inherent response limits of the materials. In contrast, magnetic bionic-structured microrobots address these hydrodynamic challenges by prioritising structural design. This class of microrobots is distinguished by complex geometric architectures that emulate the morphology or locomotion mechanisms of living organisms. Common examples include helical swimmers and artificial cilia, which enable outstanding manoeuvrability and propulsion efficiency in low-Reynolds-number fluid environments.

Bioinspired microrobots, designed to mimic the structures and movement strategies of natural organisms, demonstrate impressive motion capabilities in real-world applications [97]. The unique propulsion mechanisms of microorganisms and bacteria in the microscopic world have inspired actuation methods for microrobots [98,99]. For instance, helical microrobots, inspired by the corkscrew motion of flagellated bacteria, enable efficient propulsion in complex fluidic environments [100,101]. At the macroscopic level, the complex body structures and movement behaviours of insects and fish also serve as important references for designing the structure and function of microrobots [102].

Inspired by the bee stinger, Hu et al. designed a novel microspike robot for targeted glioma therapy [103]. As shown in Figure 4d, the microrobot was manufactured using TPP, and a hydrogel loaded with medication was applied via spin-coating to its posterior surface. The microspike structure facilitates mechanical interlocking and anchoring on the surface of the glioma. Under an alternating magnetic field, its drug release is affected by temperature. In terms of in vivo performance, the robot demonstrated excellent biocompatibility and biosafety, causing no inflammation to major organs and only minor cellular damage to the surface of brain tissue. Furthermore, in our research, we proposed the development of a fish-inspired ellipsoidal magnetic microrobot designed for multidrug delivery [104]. The microrobot comprises a magnetic-actuated robotic skeleton composed of poly(ethylene glycol) diacrylate (PEGDA) hydrogel and head and body segments composed of GelMA hydrogel, each loaded with the anticancer agents acetylsalicylic acid (ASA) and DOX, respectively. In vitro and in vivo antitumour experiments demonstrated that the ASA-DOX dual-drug-loaded microrobot exhibits superior anticancer efficacy and tumour growth inhibition compared with single-drug-loaded microrobots. Liu et al. developed a multifunctional helical microrobot with a high loading capacity and controllable release functionality [105]. As shown in Figure 4e, the microrobot was fabricated through a combination of microfluidic synthesis, polyelectrolyte complexation and surface coating with magnetic nanoparticles. In terms of propulsion speed and efficiency, the robot achieved corkscrew motion under the control of the rotating magnetic field of the 6-DOFs electromagnetic system. The robot achieved a maximum speed of 1193.42 μm/s (under an 8.5 mT magnetic field and a frequency of 7 Hz). Compared with the helical structure, Wu et al. proposed the locomotion of nonhelical multifunctional microrobots inspired by rod-shaped bacteria that can swim in helical klinotactic trajectories under rotating magnetic fields [106]. This microrobot consisted of a rigid ferromagnetic nickel head connected to a rhodium tail via a flexible, hollow hydrogel hinge based on chitosan/alginate. To adapt to different motion requirements, the microrobots can flexibly switch between tumbling, helical and asynchronous motion modes by adjusting the frequency and intensity of the rotating magnetic field [106]. Under a 15 mT magnetic field and a frequency of 30 Hz, it achieved a maximum average swimming speed of approximately 28 μm/s.

In summary, magnetic bionic-structured microrobots address the propulsion limitations of conventional carriers by mimicking the efficient locomotion strategies of natural organisms. From helical swimmers inspired by bacterial flagella to macroscopic designs mimicking fish and insects, these bio-inspired structures demonstrate superior manoeuvrability in low-Reynolds-number fluids.

### 2.4. Comparative Evaluation of Different Classifications of Magnetic Microrobots

Overall, different types of magnetic microrobots each have their own advantages in drug delivery applications. Cell-based microrobots rely on the cell’s own targeting ability and biological activity, allowing them to interact with tissues in complex physiological environments. By contrast, microalgae-based microrobots have a large surface area, which is convenient for multi-functional integration and drug loading. However, when introduced into the body, these microrobots may cause immune rejection. Advanced material-based microrobots utilise advanced material processing and manufacturing technologies to achieve a precise design of structure and function. Hydrogel materials, among them, have good biocompatibility and are suitable for drug encapsulation and stimulus-responsive release. MOF materials, with their high surface area and adjustable pore size, enable efficient drug loading and controlled release. However, the propulsion efficiency and mobility of such microrobots are often limited by the response characteristics of the materials themselves, and the preparation process requires sophisticated equipment and techniques. By contrast, bionic structural microrobots usually possess excellent mobility and navigation precision. Nevertheless, their manufacturing process is comparatively intricate, and their capacity for drug loading remains limited. In summary, future research should integrate the intrinsic characteristics of biological templates, the multifunctionality of advanced materials and the high mobility of bioinspired structures. Doing so would overcome current limitations in targeting accuracy, propulsion performance, drug loading capacity and multi-stimuli responsiveness, ultimately promoting the application of magnetic microrobots in the field of precise drug delivery.

## 3. Drug Loading Methodology of Magnetic Microrobots

The structure of microrobots not only determines their motion characteristics but is also related to drug delivery strategies. Drug delivery strategies need to consider drug loading and drug release. Drug loading involves attaching or encapsulating therapeutic drugs onto microrobot carriers using physical or chemical methods. This step is essential for microrobots to perform drug delivery applications.

### 3.1. Physical Adsorption

Physical adsorption is the process by which drug molecules adhere to the surface of microrobots through non-covalent interactions, such as van der Waals forces and electrostatic interactions [107,108]. This approach offers benefits such as operational simplicity, high tunability and controlled drug release. For macromolecular or lipophilic drugs, physical adsorption circumvents the intricacies associated with chemical modification and allows for reversible release under specific conditions, thereby enhancing drug loading efficiency [109].

**Figure 5:**
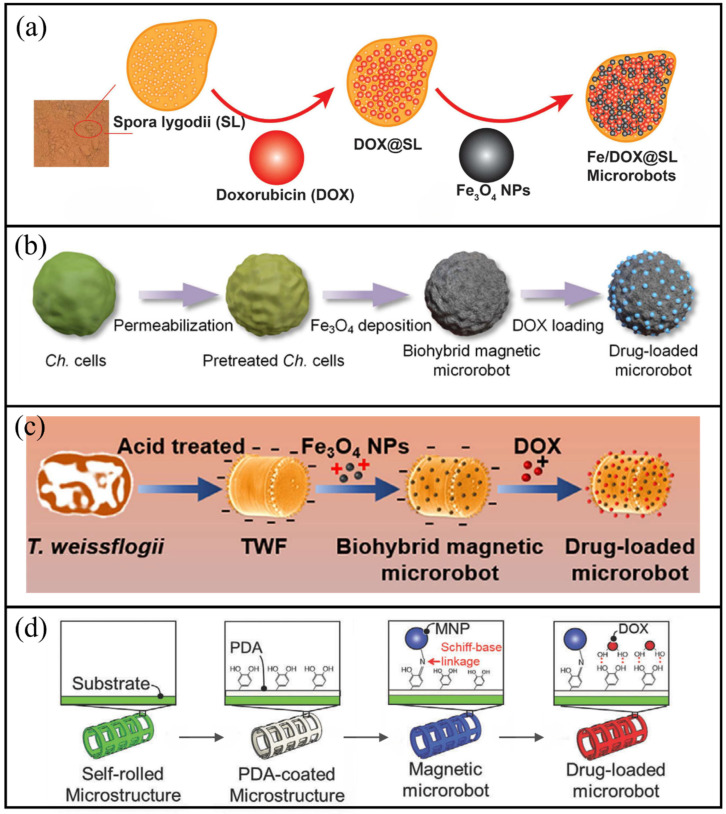
Schematic illustrations of drug loading via physical adsorption. (**a**) Adsorption of DOX molecules onto spore surfaces through electrostatic interactions. Reproduced with permission from Ref. [110]. Copyright © 2024, Open access. (**b**) Drug adsorption process on *Chlorella*-based microrobots. Reproduced with permission from Ref. [31]. Copyright © 2022, American Chemical Society. (**c**) Loading of Fe_3_O_4_ magnetic nanoparticles and DOX onto TWFs. Reproduced with permission from Ref. [65]. Copytight © 2022, Acta Materialia Inc. Published by Elsevier Ltd. All rights reserved. (**d**) Drug immobilisation on a microrobot via electrostatic adsorption. Reproduced with permission from Ref. [112]. Copyright © 2021, Wiley-VCH GmbH.

*Spora Lygodii* (SL), a traditional Chinese medicinal ingredient recognised for its therapeutic benefits, possesses a naturally porous structure that is suitable for effective drug loading. Yang et al. combined SL with microrobotic technology to fabricate Fe/DOX@SL microrobots [110]. As shown in Figure 5a, DOX was adsorbed onto the spore surface through electrostatic interactions, followed by the deposition of magnetic nanoparticles via dip-coating, resulting in the formation of the final Fe/DOX@SL microrobots. Under the guidance of an external rotating magnetic field, these microrobots can move and stay within the bladder, exhibiting prolonged mucosal adhesion and effective inhibition of tumour growth. In vitro release experiments showed that the drug released approximately 50% within 6 h and reached a cumulative release of nearly 90% after 24 h, demonstrating a sustained release characteristic. The hemolytic activity of this microrobot is extremely low (<0.1%). In vitro cell viability tests show that its cytotoxicity is lower than that of free DOX, demonstrating excellent biocompatibility. Gong et al. developed magnetic BMMs based on *Chlorella* [31]. As shown in Figure 5b, Fe_3_O_4_ nanoparticles were uniformly deposited on the surface of *Chlorella* cells (3–5 μm in diameter). The microrobots were stirred in PBS solutions with varying concentrations of DOX⋅HCl (20–160 μg/mL) to promote surface adsorption. These structures can reversibly assemble into dimers, trimers or chain-like multimers through magnetic dipole interactions and disassemble when exposed to repulsive forces. This microrobot has an extremely high drug loading efficiency, up to 98.2% (at a DOX concentration of 160 μg/mL). This outstanding loading capacity far exceeds the previous research level, fully demonstrating its advantages as an efficient drug carrier. In vitro experiments, the CCK-8 method demonstrated that its toxicity to HeLa cells was very low. Even at a concentration as high as 200 μg/mL, the cell viability still exceeded 90%. Li et al. developed a biohybrid magnetic microrobot utilising *Thalassiosira weissflogii* frustules (TWF) through the electrostatic adsorption of Fe_3_O_4_ nanoparticles and DOX [65]. As shown in Figure 5c, the microrobots, either individually or collectively, can be directed to target sites through magnetic navigation. They exhibit high drug loading capacity and pH-responsive release properties, enabling precise drug delivery to cancer cells and reducing cell viability to as low as 11.16%.

Structural design is essential in targeted drug delivery systems. Among various structures, self-rolling and folding microrobots have emerged as promising platforms for drug delivery due to their distinct movement capabilities and drug-carrying functionalities [111]. Nguyen et al. designed a magnetically guided self-rolling microrobot featuring a porous rod-like structure [112]. As shown in Figure 5d, DOX and nanoparticles were loaded on the surface of the microrobot through hydrogen bonding. The microrobot allows real-time X-ray imaging for precise positioning, and the release of DOX is triggered by the photothermal effect produced by NIR laser irradiation. After the drug is released, the microrobot can be safely recovered through an electromagnetic microrobot injector to avoid loss. Zhong et al. developed double-layered MOF-based microswimmers (DLMMs), in which anticancer drugs 5-FU and CPT-11 were loaded through electrostatic interactions [113]. DLMMs can selectively adsorb different drug combinations, showing potential to solve drug resistance. Furthermore, the drug ratio can be fine-tuned by adjusting fabrication parameters, such as the thickness of the ZIF-8 layer, thereby enhancing the adaptability of the dual-drug delivery system.

### 3.2. Covalent Bonding

Covalent bonding pertains to the process in which drug molecules establish stable bonds, such as amine linkages or disulphide bonds, with the surface of microrobots [114,115]. This method provides greater stability and a higher drug loading capacity than physical adsorption, making it ideal for long-term drug delivery applications [116].

**Figure 6:**
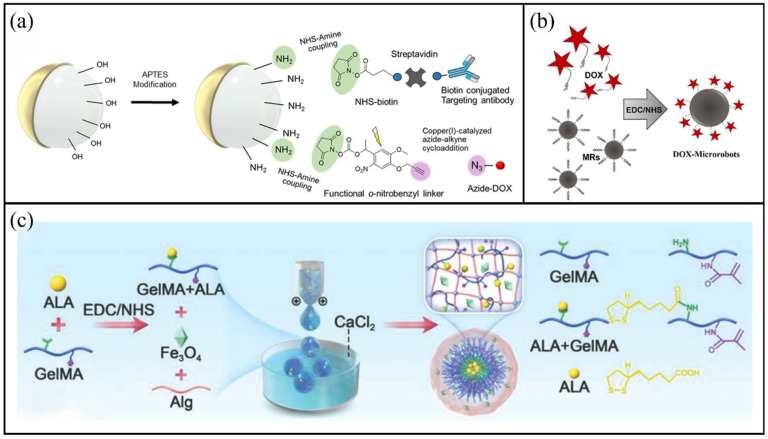
Schematic of drug loading via covalent conjugation. (**a**) Covalent conjugation of the anticancer drug DOX and targeting antibodies onto microspheres. Reproduced with permission from Ref. [117]. Copyright © 2020, The American Association for the Advancement of Science. (**b**) Covalent immobilisation of DOX on the microrobot surface. Reproduced with permission from Ref. [118]. Copyright © 2023, Wiley-VCH GmbH. (**c**) Conjugation of ALA with GelMA via chemical coupling to form a GelMA–ALA composite. Reproduced with permission from Ref. [119]. Copyright © 2023, Wiley-VCH GmbH.

Inspired by leukocytes, Alapan et al. developed multifunctional surface microrobots composed of magnetic Janus particles [117]. As shown in Figure 6a, the Janus particles were modified with targeting antibodies and photocleavable drug molecules. They were aminated using aminopropyltrimethoxysilane, followed by NHS-amine coupling chemistry to attach functional o-nitrobenzyl linkers. DOX was then conjugated to the linkers, forming stable covalent bonds. These microrobots can actively propel and steer across cell monolayers at speeds up to 600 μm/s, allowing targeted interaction with cancer cells within heterogeneous populations. Mallick et al. reported the fabrication of DOX-loaded microrobots (DOX-MRs) through covalent immobilisation [118]. As shown in Figure 6b, DOX was conjugated to magnetic beads using 1-ethyl-3-(3-dimethylaminopropyl) carbodiimide hydrochloride (EDC) and N-hydroxysuccinimide (NHS) coupling chemistry. After exposure to protease, DOX is released to induce cell apoptosis. The results show that the obtained DOX-MRs have good biocompatibility and can be effectively internalised by various types of cancer cells.

Alpha-lipoic acid (ALA), a potent antioxidant, has been used to counteract cisplatin-induced ototoxicity and hearing loss. Chen et al. developed a novel magnetic ALA-conjugated GelMA hydrogel microrobot for inner ear drug delivery and deafness prevention [119]. As shown in Figure 6c, ALA was covalently linked to amine groups on GelMA via EDC/NHS coupling, forming amide bonds (–CONH) to yield GelMA-ALA conjugates. Magnetic hydrogel microrobots were then generated using a microfluidic device, and the drug loading efficiency of ALA is 82.3%. These microrobots can be injected into the middle ear, magnetically navigated within the cavity and provide the sustained release of ALA, effectively preventing hearing loss in a cisplatin-induced deafness model. Lee et al. designed a helical magnetic microrobot based on GelMA and PEGDA, which exhibited high adaptability in viscous fluids and achieved sequential dual-drug release [120]. The initial pharmaceutical agent gemcitabine (GEM) is affixed to the surface through disulphide bonds, whereas the secondary drug DOX is enclosed within the matrix. In terms of in vitro performance, this system, in the HuCCT1 liver cancer sphere model, achieved the separation and recovery of MNP (with a separation efficiency of 94.0%) and sequential dual-drug release through an integrated system. When GEM was actively released and DOX was subsequently released, the best therapeutic effect was achieved, with approximately 70% of the target cancer cells being cleared.

### 3.3. Encapsulation Method

Encapsulation refers to the process of enclosing drug molecules within a carrier material, either physically or chemically, to form a protective structure [121]. Compared with physical adsorption and covalent conjugation, encapsulation has a higher drug loading capacity, enhances the protection of the therapeutic agents from environmental degradation and can achieve sustained and controllable release by tuning the properties of the encapsulating material [122].

**Figure 7:**
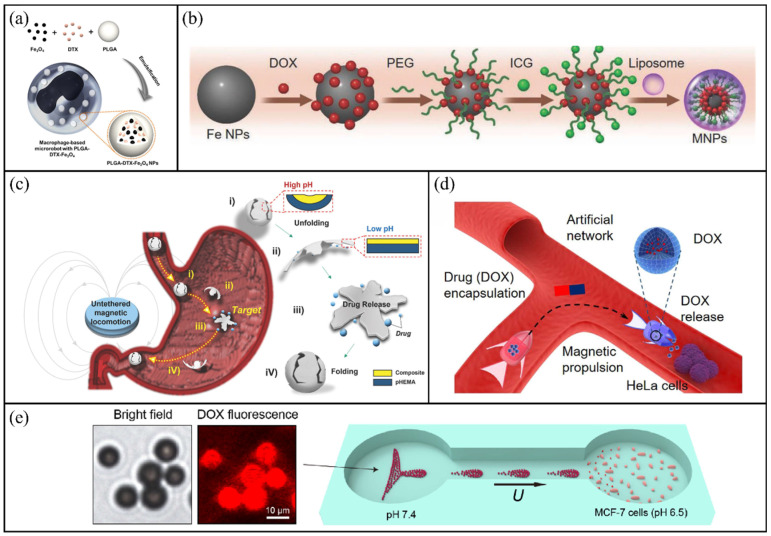
Schematic illustrations of drug encapsulation and pH-responsive microrobots. (**a**) Macrophage-based microrobot loaded with PLGA-DTX-Fe_3_O_4_ nanoparticles. Reproduced with permission from Ref. [123]. Copyright © 2016, Open access. (**b**) Fabrication process of GelMA-based microrobots. Reproduced with permission from Ref. [124]. Copyright © 2021, Wiley-VCH GmbH. (**c**) Deformation behaviour of a pH-responsive bilayer microrobot under different pH conditions. Reproduced with permission from Ref. [129]. Copyright © 2022, Royal Society of Chemistry. (**d**) Schematic illustration of magnetic SMMFs achieving targeted DOX release for cancer treatment through shape transformation. Reproduced with permission from Ref. [130]. Copyright © 2021, American Chemical Society. (**e**) Targeted drug delivery to tumours using collectively moving PC-bots inside a microfluidic channel. Reproduced with permission from Ref. [131]. Copyright © 2023, Open access.

Han et al. developed macrophage-based magnetic microrobots for targeted cancer therapy [123]. As shown in Figure 7a, DOX and Fe_3_O_4_ nanoparticles were encapsulated into poly(lactic-co-glycolic acid) (PLGA) to form mixed nanoparticles, which were subsequently internalised by macrophages via endocytosis, resulting in functional cell-based microrobots. These microrobots utilise the intrinsic cancer-targeting capability of macrophages to infiltrate tumour tissues and deliver encapsulated therapeutic agents, thereby inhibiting cancer cell proliferation. Similarly, Dai et al. engineered macrophage-based microrobots for light-controlled drug delivery [124]. As shown in Figure 7b, liposomal nanoparticles (MNPs) composed of iron nanoparticles (FeNPs), DOX and the photothermal molecule indocyanine green were encapsulated into macrophages through endocytosis.

Unlike previous systems, these microrobots enable cascaded drug release upon exposure to NIR light, reaching up to 97% effectiveness in destroying cancer cells. Tian et al. proposed an enzyme-responsive encapsulation strategy using core–shell structured magnetic microrobots (ChemoBots) for targeted drug delivery [125]. The outer shell, fabricated through TPP of PEGDA, permits the penetration of matrix metalloproteinases (MMP2/MMP9), which are prevalent in the tumour environment. The inner core is made of GelMA hydrogel that physically encapsulates DOX and Fe_3_O_4_ nanoparticles. Upon reaching the tumour site, MMP-mediated degradation of the GelMA network triggers sustained drug release for up to 357 h, significantly suppressing growth in triple-negative breast cancer models. Qiao et al. developed a hydrogel-based magnetic capsule microrobot for intravascular targeted drug delivery using a tri-axial microfluidic device [126]. An indomethacin solution was administered as the internal phase of the microrobot, including an intermediate layer that comprised sodium alginate, CaCO_3_ and Fe_3_O_4_ nanoparticles. The outer oil phase contained acetic acid, which triggered Ca^2+^ release from CaCO_3_ upon contact, resulting in the rapid cross-linking of alginate at the outlet to form a hydrogel shell that encapsulated the drug. The embedded magnetic particles enable the guided locomotion of the microrobot under external magnetic fields, offering drug protection, sustained release and precise localised delivery.

### 3.4. Comparative Analysis of Drug Loading Methods

In summary, the drug loading strategies of magnetic microrobots mainly include physical adsorption, covalent bonding and encapsulation. Physical adsorption uses non-covalent interaction to realise the convenient binding of the drug with the surface, so as to maintain the biological activity of the therapeutic drug. However, due to its dependence on weaker van der Wals forces or electrostatic electricity, it often leads to poor stability, and drugs are prone to premature leakage during transportation, limiting their application in the delivery of highly toxic drugs. On the contrary, covalent bonding can provide a strong chemical connection to ensure the high stability of the drug and prevent off-target release. However, the method also has some scientific limitations, such as the fact that chemical modification may affect the efficacy of the drug, and the limited load of the available surface functional group density.

In terms of drug loading efficiency, encapsulation is generally considered a better method because it uses the internal volume of the carrier to achieve high load capacity and protect the drug from enzyme degradation. However, the envelope faces many challenges in achieving precise release kinetics, and there are often initial “sudden release” or diffusion resistance in the matrix. In addition, the choice of drug-loading method is closely related to the size and geometry of the microrobot. Microrobots with high surface area volume ratio or porous structures, such as MOFs and diatom shells, are very suitable for physical adsorption. On the contrary, microrobots with three-dimensional hydrogel networks or hollow internal structures are very suitable for packaging, so that they can store a large number of drugs. For rigid structures with limited internal volume, covalent bonding is still the best strategy to ensure drug retention. In the end, a critical gap remains in developing hybrid strategies that combine the high capacity of encapsulation with the stability of covalent bonding, without necessitating complex, multi-step fabrication processes that hinder clinical scalability.

## 4. Drug Release Methodology of Magnetic Microrobots

After the drugs are delivered to the target site of the lesion, they must be released to achieve the treatment effect. The drug release strategy influences the duration of release and the effectiveness of drug administration. Therefore, controlling the drug release is also a crucial aspect for achieving effective targeted therapy. Drug release methods are related to the methods in which drugs are loaded; however, there are distinctions among them. In this section, we have summarised and analysed the various mechanisms of drug release and their therapeutic effects.

### 4.1. pH-Responsive Release

The pH-responsive release strategy utilises the pH changes in different tissues or pathological sites to achieve selective drug release in specific microenvironments [127]. This mechanism is especially beneficial in regions with significant pH gradients, such as tumour tissues or the gastrointestinal tract, as it improves drug targeting and reduces systemic side effects [128].

Sodium alginate is a well-known pH-sensitive material, and microrobots made from alginate hydrogels undergo swelling and shrinking transitions under various pH conditions. Jia et al. developed mitomycin-loaded magnetic-driven hydrogel microrobots (MHRMs) for bladder cancer therapy [89]. These microrobots feature a pH-responsive release mechanism designed to adapt to the bladder microenvironment. They maintain structural integrity and achieve stable drug release in the weakly acidic urine typical of bladder cancer patients (pH 6.5), while undergoing rapid degradation in simulated normal urine (pH 6.8). Furthermore, guided by an external magnetic field, MHRMs execute spiral locomotion along the bladder wall, ensuring uniform drug distribution. In addition to alginate, poly(2-hydroxyethyl methacrylate) (pHEMA) hydrogel is presented as another pH-responsive system, distinguished by its excellent biocompatibility and tissue-mimicking physical properties. Darmawan et al. developed a magnetically controlled bilayer microrobot with pHEMA as the pH-sensitive outer layer and a composite resin containing X-ray contrast agents as the inner core [129]. As illustrated in Figure 7c, fabricated through 3D printing, these compact microrobots remained folded in high-pH conditions and unfolded within acidic gastric environments, facilitating the release of the encapsulated drug. Moreover, in an acidic environment (pH 5.5), the release kinetics was faster. Within 48 h, 78.9% of the total drug dose was released in the pH 5.5 environment, while only 45.9% was released in the pH 7.4 environment. Notably, the use of contrast agents enabled real-time X-ray tracking, and cytotoxic effects were confirmed against cancer cells.

Based on the concept of shape deformation, Xin et al. utilised 3D laser printing technology to construct environment-adaptive magnetic microrobots with heterogeneous structures derived from a single pH-sensitive hydrogel [130]. As illustrated in Figure 7d, the mouth of the microrobot stayed closed in neutral conditions (pH 7.4) to hold drugs and opened on its own in mildly acidic environments (pH < 7) to release drugs. Operated under external magnetic fields, these microrobots successfully localised to tumour sites and harnessed the acidic microenvironment to initiate targeted drug delivery, achieving precise therapeutic intervention. Unlike the previous examples that emphasise structural deformation for release, Yu et al. proposed a magnetic-photonic-crystal microrobot swarm composed of Fe_3_O_4_ nanoparticles and integrated pH-responsive hydrogel photonic crystals [131]. As shown in Figure 7e, under acidic conditions, the shrinkage of the hydrogel microswarm induces reversible changes in the photonic lattice spacing, resulting in instantaneous structural colour variation for visual pH detection. Simultaneously, the swelling and shrinking behaviours of the hydrogel enables adaptive drug release. At a physiological pH of 7.4, only 14.2% of DOX was released within 4 h. In contrast, under acidic conditions (pH 6.5 and pH 5.0), the cumulative release significantly increased to 21.7% and 53.3%, respectively.

### 4.2. Hydrolysis Release

In microrobotic drug delivery applications, hydrolysis pertains to the utilisation of hydrolysable chemical bonds within the microrobot’s material (such as ester or peptide bonds) that cleave under specific environmental conditions, resulting in structural disintegration and subsequent drug release [132]. This strategy requires no external stimuli, relying solely on physiological factors such as pH levels, enzyme concentrations, or temperature changes within the body, which offers excellent biocompatibility and predictable release behaviour [133,134].

**Figure 8:**
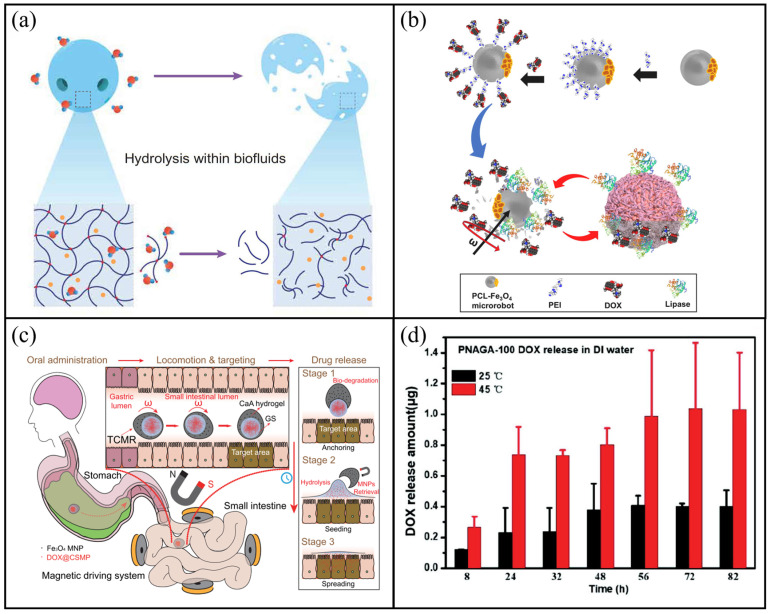
Biodegradation-responsive microrobots. (**a**) Schematic of BAM hydrolysis. Reproduced with permission from Ref. [135]. Copyright © 2024, The American Association for the Advancement of Science. (**b**) Schematic illustration of enzyme-mediated biodegradation of PCL-Fe_3_O_4_/PEI@DOX magnetic microrobots inducing drug release and cancer cell death. Reproduced with permission from Ref. [136]. Copyright © 2022, Elsevier Ltd. All rights reserved. (**c**) Drug release mechanism of a microrobot encapsulated with a thermo-responsive biocompatible fatty acid-based mixture. Reproduced with permission from Ref. [137]. Copyright © 2021, American Chemical Society. (**d**) DOX release of PNAGA-100 at 25 °C and 45 °C. Reproduced with permission from Ref. [138]. Copyright © 2023, Open access.

A collaborative team from the California Institute of Technology and the University of Southern California developed a novel biodegradable hydrogel microrobot (BAM), which demonstrates efficient navigation in complex biological environments and successful drug delivery [135]. The main body of BAM consists of PEGDA, a biodegradable material that reduces the risk of post-delivery residue. As shown in Figure 8a, upon reaching the target site via magnetic guidance, the microrobot initiates drug release through biodegradation. The release kinetics consist of two distinct stages: an initial rapid release phase where approximately 60% of the drug is released within 12 h, primarily driven by diffusion, followed by a sustained, slower release phase governed by polymer degradation. Notably, the release kinetics are influenced by molecular size. The larger model drug (Rhodamine B) exhibited a comparatively slower rate, requiring approximately 19 h to achieve 60% cumulative release. Pacheco et al. proposed a different enzyme-triggered magnetically controlled microrobot for targeted drug delivery, which achieves sustained release through programmed self-destruction [136]. The microrobot employs poly(ε-caprolactone) (PCL) as a biodegradable structural matrix and demonstrated excellent biocompatibility, with no significant toxicity observed in HeLa cells. As shown in Figure 8b, the system targets pancreatic cancer cells by leveraging their overexpression of lipase, which preferentially degrades the PCL shell. This leads to autonomous disintegration of the microrobot at the disease site and subsequent drug release, enabling controlled and targeted therapy. This enzyme-mediated mechanism ensures that drug release occurs specifically within the pathological microenvironment, combining targeting precision with high biocompatibility. Chen et al. developed a triple-configuration magnetic microrobot (TCMR) that demonstrates a highly sophisticated multi-component cooperative degradation and release strategy [137]. The system comprises three coaxial nested structures: a drive and protection unit, an anchoring and seeding unit and a drug release unit. As shown in Figure 8c, the TCMR can be accurately navigated to the target site via a magnetic guidance system. Subsequently, the drive and protection unit disintegrate due to the dissolution of the intestinal pH-responsive material. Thereafter, gelatine microspheres within the drug release unit undergo gradual degradation through protease-mediated enzymatic hydrolysis, resulting in the slow and controlled release of the loaded drugs. This multi-stage process enables the exact and targeted delivery of drugs.

### 4.3. Temperature-Responsive Release

Temperature-responsive release is another strategy for on-demand drug release in microrobotic systems. This approach relies on the phase transition or volumetric alterations, such as swelling and shrinking, of thermoresponsive materials in response to temperature variations, particularly in the vicinity of human body temperature (37 °C), to regulate drug delivery release.

Poly-N-acryloyl glycinamide (PNAGA) is a thermosensitive hydrogel that undergoes pronounced swelling at approximately 45 °C. Using TPP technology, Zhou et al. fabricated PNAGA-based microrobots capable of thermally triggered release [138]. As shown in Figure 8d, the PNAGA hydrogel matrix remained contracted at room temperature (25 °C), maintaining drug encapsulation. Upon exposure to near-physiological hyperthermia (≈45 °C), the hydrogel network rapidly swelled, resulting in the release of the encapsulated drug. Notably, the PNAGA-100 variant exhibited the most significant response, achieving a rapid expansion rate of 22.5% at 45 °C. Drug release tests confirmed that the drug release of these thermosensitive microrobots at 45 °C was 2 to 3 times higher than that at 25 °C, highlighting their potential for targeted drug delivery in the human body. Unlike the swelling mechanism based on polymers, Chen et al. developed a fillable magnetic microrobot for cardiac drug delivery [139]. In this design, the cavity of the microrobot was filled with therapeutic agents and sealed at the top by a thin film of fatty acids. At temperatures below physiological levels, the fatty acid film remained solid, effectively preventing drug leakage. However, upon exposure to body temperature (37 °C), the fatty acid layer underwent solid-to-liquid phase transition, gradually opening the cavity pores and allowing for the continuous and controlled release of the encapsulated drugs. Quantitative analysis confirmed the efficacy of this sealing strategy in preventing premature leakage. While unsealed microrobots exhibited a rapid burst release of approximately 85.5% within just 10 min, the fatty acid-sealed counterparts significantly extended the release window. Specifically, over a 2 h period, the cumulative release was modulated between 70.70% and 81.88% depending on the fatty acid ratio, demonstrating the system’s capability to achieve sustained and tuneable drug delivery profiles under physiological conditions.

### 4.4. Magnetic-Controlled Release

Magnetic fields are widely favoured in drug delivery microrobotic systems due to their safety and minimal harm to biological tissues. Xu et al. designed a soft capsule microrobot composed of magnetic nanoparticles dispersed in a flexible hydrogel matrix [140]. An external magnetic field can direct the microrobot, while pulsed magnetic fields induce localised deformation or rupture of the capsule structure, thereby enabling the rapid release of encapsulated drugs suitable for high-concentration and localised delivery. Ziegler et al. highlighted the need for remotely adjustable release strategies in implantable drug delivery systems [141]. By embedding magnetic nanoparticles and drug molecules uniformly within electrospun polymer fibres, they demonstrated that the particles generate localised magnetothermal effects under an alternating magnetic field. The heat induces enhanced chain mobility or partial melting of the polymer matrix, accelerating drug diffusion and release. In the absence of the magnetic field, drug efflux is significantly reduced, allowing programmable release profiles.

Amirov et al. proposed a magnetically triggered phase-transition material for accurate heat-controlled release of drugs [142]. They fabricated composite particles by mixing iron alloy nanoparticles with thermo-sensitive polymer PNIPAM, in which the iron alloy nanoparticles exhibit magnetothermal effects. Under magnetic stimulation, the FeRh nanoparticles shift to a ferromagnetic phase, emitting heat, thereby inducing the volume phase transition temperature of PNIPAM. This mechanism enables the controlled release of DOX, combining magnetothermal response and temperature sensitivity to achieve low background leakage and high triggering efficiency in deep tissues. Kim et al. developed a bilayer hydrogel microrobot consisting of a magnetic nanoparticle layer and a therapeutic layer for treating ocular diseases [143]. The microrobot was transported to the target site using an externally applied magnetic field. When an AMF is applied at the target site, the MNPs generate localised heat, which triggers the dissolution of the treatment layer. Following the completion of drug delivery, the driver layer containing the MNPs (PEGDA core) is retrieved using an external magnetic field.

### 4.5. Light-Triggered Release

Light-triggered drug release has become a promising targeted delivery method that uses the photothermal or photochemical effects of light at specific wavelengths to control drug release from carriers [144]. This approach offers high tissue penetration and minimal damage, demonstrating significant potential in applications such as cancer therapy and tissue regeneration [145].

**Figure 9:**
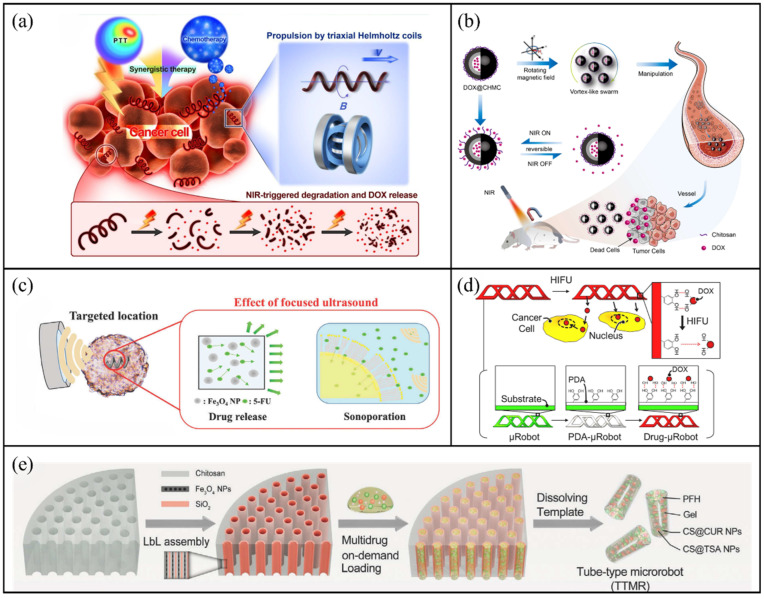
Microrobots based on light-triggered and ultrasound-mediated release. (**a**) Schematic illustration of magnetic microrobot propulsion, NIR-triggered degradation, DOX release and the mechanism of combined chemo-photothermal therapy against cancer cells. Reproduced with permission from Ref. [30]. Copyright © 2019, American Chemical Society. (**b**) Magnetically driven targeted drug delivery using a CHMC microrobot swarm. Reproduced with permission from Ref. [147]. Copyright © 2024, American Chemical Society. (**c**) In vitro demonstration of on-demand drug release triggered by ultrasound. Reproduced with permission from Ref. [150]. Copyright © 2020, Elsevier B.V. All rights reserved. (**d**) Conceptual diagram of ultrasound-mediated drug delivery using PDMs. Reproduced with permission from Ref. [149]. Copyright © 2020, Wiley-VCH GmbH. (**e**) TTMRs made with the assistance of templates. Reproduced with permission from Ref. [151]. Copyright © 2024, Wiley-VCH GmbH.

Wang et al. utilised *Spirulina* as a natural helical template and enhanced its functionality by depositing core–shell Pd@Au nanoparticles on its surface through electroless plating [30]. An electroless deposition method was used to coat the surface of the helical template with core–shell Pd@Au nanoparticles to enhance photothermal conversion, whereas Fe_3_O_4_ nanoparticles were incorporated via a sol–gel process to enable magnetic actuation. As shown in Figure 9a, the system exhibited a high propulsion efficiency of 526.2 μm/s under a rotating magnetic field and disintegrated into individual particles upon NIR laser irradiation, facilitating drug release. This design demonstrated superior therapeutic efficacy through the combination of chemotherapy and photothermal therapy compared with single-modality systems.

Addressing challenges in controlled drug release during skin tissue repair, Sun et al. designed a gelatine–hesperidin composite microrobot that integrates magnetic navigation and light-responsive drug release [146]. Fabricated using microfluidic technology, these microrobots incorporated photosensitive groups within the gelatine hydrogel matrix. Upon exposure to light of a specific wavelength, local photothermal effects induced the relaxation of the hydrogel network, accelerating drug diffusion. And in vitro drug release experiments revealed that approximately 78% of the drug was released within 30 min. Chen et al. further expanded the scope of light-responsive systems by developing a hollow magnetic nanocarrier-based microrobot swarm responsive to NIR irradiation [147]. The drug (DOX) is encapsulated in the cavity of a hollow porous carbon shell. Surface-modified chitosan acts as an NIR responsive molecular valve. As shown in Figure 9b, NIR irradiation causes CHMC to generate heat, increasing the distance between chitosan molecules, weakening electrostatic interactions, and thereby enhancing the movement of DOX molecules, achieving reversible Nir-responsive release.

### 4.6. Ultrasound-Mediated Release

Ultrasonic waves, due to their low absorption rate and high efficiency in biological media, can be used as a non-contact and safe method for advancing and manipulating microrobots [148]. Ultrasound stimulation has the capacity to initiate drug release via cavitation effects and acoustic streaming. It may also alter tissue permeability and absorption properties, facilitating drug penetration into cells and enhancing therapeutic efficacy, particularly in cancer treatment.

Park et al. developed porous degradable microrobots (PDMs) incorporating magnetite nanoparticles and the anticancer drug 5-fluorouracil (5-FU) [149]. As shown in Figure 9c, ultrasound exposure induced cavitation and acoustic streaming, promoting efficient drug release. By adjusting ultrasound beam parameters, the system achieved versatile release profiles, including herein as natural, burst, and constant. In the burst release mode, the drug release rate is 2.75 times that of the natural release mode (4.39 ng vs. 1.6 ng per hour). Similarly, Darmawan et al. proposed a self-folding helical microrobot drug delivery platform that utilises magnetic fields for navigation, followed by ultrasound stimulation for triggered release [150]. As shown in Figure 9d, anticancer drugs were bound to the microrobot surface via noncovalent interactions. Upon short-term ultrasound exposure (≈approximately 1 min), over 90% of the drug payload was released, rapidly achieving therapeutic concentrations and significantly enhancing treatment efficacy. Beyond nanoparticle-based carriers, phase-change materials such as perfluorohexane (PFH) have been introduced to ultrasound-responsive systems. Under external ultrasound irradiation, PFH undergoes a liquid-to-gas phase transition, generating mechanical forces that can disrupt biological barriers and trigger on-demand release. As shown in Figure 9e, Yi et al. designed tube-type microrobots (TTMRs) capable of penetrating the round window membrane for inner ear therapy [151]. TTMRs were loaded with multiple therapeutic agents, including curcumin, tanshinone IIA (TSA) and PFH. Upon ultrasound stimulation, the vaporisation of PFH produced a ‘micro-shotgun’ effect that propelled drugs across the membrane into the inner ear. This multi-drug delivery system allows for precise dosage ratio adjustments. Quantitative studies confirmed that release efficiency is controllable via excitation voltage and duty cycle, offering a broad adjustment range (10–100%). By optimising parameters to 4.5 V and 50% duty cycle, the authors achieved approximately 90% drug release while ensuring the acoustic intensity remained below the biological safety threshold of 3 W cm^−2^, demonstrating both high efficiency and biocompatibility.

### 4.7. Multiple Response System

Multistimuli-responsive release systems integrate two or more environmental or external trigger mechanisms to achieve precise control over drug delivery and enable synergistic therapeutic effect [152]. These systems usually combine physical actuation fields, such as magnetic fields, light and ultrasound, with chemical or biological signals, including pH, temperature or enzymatic activity. This integration not only enhances spatiotemporal control over release kinetics but also improves adaptability to complex in vivo microenvironments, thereby increasing efficiency and flexibility in precision medicine [153].

Demonstrating the versatility of biohybrid systems, Akolpoglu et al. engineered a bacterial microrobot capable of responding to both endogenous and exogenous stimuli [154]. Through the integration of drug-loaded nanoliposomes, the system utilises pH variations for passive, long-term release, releasing 98% of the payload in highly acidic conditions (pH = 2.5), while employing NIR light for active, on-demand intervention. The NIR stimulation not only triggered a rapid drug release (approximately 50% within 5 h) via photothermal membrane disruption but also served as a biocontainment mechanism, ensuring the controlled elimination of the living bacteria after therapeutic action. Yang et al. developed biodegradable helical microrobots (MXBOTs) by integrating GelMA hydrogels with Ti_3_C_2_ MXene nanosheets and Fe3O4 nanoparticles [82]. DOX was loaded onto the MXene surface via electrostatic interactions (loading efficiency approximately 69%). The system employs a synergistic pH/thermal dual-responsive release strategy. In the acidic tumour microenvironment (pH 5.6), the protonation of hydroxyl groups on the Ti_3_C_2_ surface weakens the electrostatic binding with DOX, while localised hyperthermia (50 °C) further disrupts these interactions and accelerates molecular diffusion. This mechanism ensures a sustained release profile (35.0% over 24 h) without initial burst effects. Gu et al. engineered a MOF-functionalized biohybrid microrobot by integrating *Chlorella* cells with a PDA coating and ZIF-8 nanoparticles [96]. This system achieved an exceptional drug loading efficiency of approximately 99.8% via electrostatic adsorption. Crucially, the cargo release is driven by a synergistic pH/NIR dual-trigger mechanism. The acidic tumour microenvironment (pH = 5.4) induces the protonation and structural collapse of the pH-sensitive ZIF-8 framework, while NIR irradiation generates localised hyperthermia via the PDA layer. This thermal energy accelerates release by disrupting the coordination bonds between the drug molecules and the ZIF-8 lattice, thereby ensuring precise, high-concentration drug delivery specifically at the tumour site.

### 4.8. Comparative Analysis of Drug Release Methods

Different drug loading methods and release strategies are closely connected in the design of drug delivery systems, and their combined effects influence the delivery efficiency and therapeutic result. To provide a clear overview, Table 1 outlines the main drug loading methods and release mechanisms used in magnetic microrobot-based delivery systems, including their principles, advantages, limitations, and unique features. In terms of drug release, a critical analysis reveals a distinct trade-off between internal biological triggers and external physical stimuli, which dictates their clinical adaptability. Internal stimuli-responsive strategies, primarily pH-responsive and enzyme-degradable mechanisms, offer the advantage of autonomous activation within the pathological microenvironment. These methods are clinically well-suited for providing continuous, sustained release profiles necessary for long-term tumour suppression. However, their precision is often limited by the physiological heterogeneity between patients and local fluctuations in pH or enzyme levels. Conversely, external stimuli-responsive strategies, such as light, ultrasound, and magnetic actuation, provide superior spatiotemporal control, allowing clinicians to precisely modulate the “on-demand” release. However, their clinical applications are strictly limited by the depth of physical penetration. Although the light trigger system has high accuracy, they are limited to superficial or endoscopic applications due to poor tissue penetration. In contrast, magnetic and ultrasound modalities can penetrate deep tissues, making them adaptable for internal organ targeting. Regarding release kinetics, the ultrasound-mediated strategy is recognised as the method with the fastest release rate. By utilising cavitation effects or phase-change materials, it can trigger a rapid “burst” release of the payload within minutes. However, in many drug delivery scenarios, particularly cancer treatment, a continuous and sustained release profile is often required to maintain therapeutic concentrations over time and fully exert the drug’s efficacy. Therefore, balancing the need for rapid acute intervention (via ultrasound) with the need for long-term maintenance (via degradation or diffusion) remains a critical design consideration. Consequently, multi-responsive strategies that integrate the specificity of external triggers with the sustained nature of internal stimuli represent a vital direction for improving targeting capability and therapeutic outcomes in complex biological environments.

## 5. Conclusions and Future Perspectives

Magnetic microrobots have emerged as promising platforms for targeted drug delivery due to their ability to be remotely actuated and precisely controlled. Compared with conventional drug delivery approaches, magnetic microrobots offer several advantages, including accurate manipulation, rapid response, and high drug-loading capacity, thereby exhibiting significant potential in precision medicine and personalised therapy. In this review, we systematically summarise the latest advancements in the fabrication of drug delivery magnetic microrobots, including biomimetic, bio-template, and advanced material-based magnetic microrobots, and evaluate their respective advantages and limitations. We focus on introducing the key drug loading and release strategies of magnetic microrobots, such as physical adsorption, covalent bonding, encapsulation, and multi-stimuli response mechanisms.

Overall, research on magnetic microrobots for drug delivery is progressing toward intelligent, biocompatible, and biodegradable systems through innovations in material selection, structural design, and loading/release methodologies. With the development of magnetically responsive, biodegradable, flexible, and multifunctional integrated materials, microrobots are expected to maintain stable propulsion in complex physiological environments while meeting stringent safety requirements for biomedical applications. Meanwhile, drug-delivery needs are shifting from single-agent administration toward multi-drug synergistic therapy, targeted molecular integration, multi-stimuli-responsive actuation, and precisely controlled release.

Despite the encouraging results obtained from in vitro studies and preliminary in vivo experiments in small animal models, significant challenges remain before the clinical translation of drug delivery magnetic microrobots can be achieved. Critical issues such as immunogenicity, long-term biosafety, biodegradability, and in vivo clearance pathways are still insufficiently understood and lack standardised evaluation protocols. In realistic physiological environments, microrobots must operate in dynamic and heterogeneous conditions, where protein corona formation, immune recognition, non-Newtonian fluid behaviour, and biological barriers may significantly alter propulsion efficiency, targeting accuracy, and drug release performance. Consequently, the transition from controlled in vitro validation to reliable in vivo application remains the principal bottleneck for clinical implementation.

Beyond biological constraints, the transformation and commercialization of biomedical microrobots face substantial technological and regulatory challenges. Current fabrication techniques often involve complex processes, high costs, and limited scalability, resulting in poor reproducibility and inconsistent magnetic responsiveness, factors which restrict large-scale manufacturing and clinical testing. Furthermore, the multifunctional nature of microrobots, combining features of drug carriers, medical devices, and actively propelled systems, creates ambiguity in regulatory classification, potentially prolonging approval timelines and increasing commercialization risks.

Future research in magnetic microrobot-based drug delivery is therefore expected to focus on the development of highly biocompatible and biodegradable magnetically responsive materials, such as degradable metal–organic frameworks and magnetic bio-based hydrogels, together with rational structural designs that support programmable drug loading and controlled release. In parallel, advances in microfluidics, high-resolution 3D printing, and scalable self-assembly technologies are anticipated to improve manufacturing throughput, structural consistency, and functional reliability. Collectively, these interdisciplinary advances will lay the essential groundwork for translating magnetic microrobots from experimental proof-of-concepts toward clinically viable platforms for targeted drug delivery, ultimately enabling their application in precision and personalised medicine. 

## Figures and Tables

**Table 1:** Summary and comparison of drug loading and release strategies.

Category	Classification	Principle	Advantages	Limitations	Features	Drug Loading Efficiency	Release Rate	Reference
Drug Loading	Physical adsorption	Weak interactions (electrostatic, van der Waals, H-bonds)	Simple preparation, mild toward drug activity	Weak stability, potential premature release	Easy to implement, preserves drug activity	low	/	[65,110,112,113]
	Covalent conjugation	Covalent bonding between drug and carrier functional groups	High stability, programmable release	Requires chemical modification, may affect drug activity	Strong binding, precise release control	moderate	/	[117,118,119,120]
	Encapsulation	Drugs confined within hydrogels, vesicles, MOFs, or porous shells	High drug loading protects drugs from environment	Fabrication complexity varies	Protects drugs, enables sustained or controlled release	High	/	[123,124,125,126]
Drug Release	pH-responsive	Acid-triggered degradation or bond cleavage	Tumour targeting, selective release	Sensitive to pH fluctuations	Exploits tumour microenvironmet	/	Moderate	[89,129,130,131]
	Hydrolysis-release	Hydrolysable linkers or matrices degrade in aqueous environments	Predictable release kinetics	Hydrolysis rate depends on local environment	Biodegradable materials, controlled release	/	Slow	[135,136,137]
	Temperature-responsive	Thermo-induced phase transition or thermal breakdown of matrix	On-demand release, compatible with hyperthermia	Requires local or external heating	Thermo-sensitive carriers, hyperthermia-assisted	/	Moderate	[138,139]
	Magnetic-controlled release	Magnetothermal heating or magnetic-force-triggered deformation	Remote control, deep tissue penetration	Equipment needed, potential thermal safety concerns	Magnetic actuation or heating	/	On-demand /tuneable	[140,141,142,143]
	Light-triggered release	Photothermal/photocleavage-driven release through illumination	High spatial/temporal precision	Limited tissue penetration	Precise spatiotemporal control	/	Fast/on-demand	[30,146,147]
	Ultrasound-mediated release	Cavitation, acoustic heating, mechanical disruption	Non-invasive, deep penetration	Risk of tissue damage under high intensity	Deep-tissue delivery, non-invasive	/	Fast	[149,150,151]
	Multiple-response	Integration of pH, thermal, light, enzymatic, or magnetic triggers	High specificity, enhanced precision	Complex design and fabrication	Intelligent, multi-triggered release	/	Tuneable	[82,96,154]

## Data Availability

No new data were created or analyzed in this study.
